# National registry for amyotrophic lateral sclerosis: a systematic review for structuring population registries of motor neuron diseases

**DOI:** 10.1186/s12883-021-02298-2

**Published:** 2021-07-06

**Authors:** Ingridy Barbalho, Ricardo Valentim, Mário Dourado Júnior, Daniele Barros, Hércules Pedrosa Júnior, Felipe Fernandes, César Teixeira, Thaísa Lima, Jailton Paiva, Danilo Nagem

**Affiliations:** 1 Laboratory of Technological Innovation in Health (LAIS), Federal University of Rio Grande do Norte (UFRN), Natal, Brazil; 2 Department of Integrated Medicine, Federal University of Rio Grande do Norte (UFRN), Natal, Brazil; 3 Univ Coimbra, Centre for Informatics and Systems of the University of Coimbra, Department of Informatics Engineering, Coimbra, Portugal; 4 Brazilian Ministry of Health, Brasília, DF Brazil; 5 Federal Institute of Rio Grande do Norte, Natal, Brazil

**Keywords:** Amyotrophic lateral sclerosis, Rare disease registries, Motor neuron disease, National databases of epidemiological, Global health

## Abstract

**Background:**

This article comprises a systematic review of the literature that aims at researching and analyzing the frequently applied guidelines for structuring national databases of epidemiological surveillance for motor neuron diseases, especially Amyotrophic Lateral Sclerosis (ALS).

**Methods:**

We searched for articles published from January 2015 to September 2019 on online databases as PubMed - U.S. National Institutes of Health’s National Library of Medicine, Scopus, Science Direct, and Springer. Subsequently, we analyzed studies that considered risk factors, demographic data, and other strategic data for directing techno-scientific research, calibrating public health policies, and supporting decision-making by managers through a systemic panorama of ALS.

**Results:**

2850 studies were identified. 2400 were discarded for not satisfying the inclusion criteria, and 435 being duplicated or published in books or conferences. Hence, 15 articles were elected. By applying quality criteria, we then selected six studies to compose this review. Such researches featured registries from the American (3), European (2), and Oceania (1) continent. All the studies specified the methods for data capture and the patients’ recruitment process for the registers.

**Discussions:**

From the analysis of the selected papers and reported models, it is noticeable that most studies focused on the prospect of obtaining data to characterize research on epidemiological studies. Demographic data (ID01) are present in all the registries, representing the main collected data category. Furthermore, the general health history (ID02) is present in 50% of the registries analyzed. Characteristics such as access control, confidentiality and data curation. We observed that 50% of the registries comprise a patient-focused web-based self-report system.

**Conclusion:**

The development of robust, interoperable, and secure electronic registries that generate value for research and patients presents itself as a solution and a challenge. This systematic review demonstrated the success of a population register requires actions with well-defined development methods, as well as the involvement of various actors of civil society.

## Background

Amyotrophic Lateral Sclerosis (ALS) is a nervous system disease considered rare, degenerative, incapacitating and that is irreversible thus far [1–5]. ALS is characterized by the degeneration of the motor neuron at several levels: bulbar, cervical, thoracic, and lumbar [6, 7]. The worldwide incidence of ALS ranges from 0.6 to 3.8 cases per 100,000 people per year, whereas the prevalence is approximately 4.1 to 8.4 per 100,000 [8]. Furthermore, cases of ALS are projected to globally increase from 222,801 in 2015 to 376,674 in 2040, representing an upsurge of 69% [9]. Such a fact is related to population aging, particularly among developing countries [10].

Due to its progression, approximately 50% of patients diagnosed with ALS have a life expectancy of 30 months after the onset of symptoms, and only about 10% of patients survive for more than a decade. [10]. Aiming to increase the life expectancy of ALS patients, it is essential to formulate therapies that not merely reduce the disease progression, but also are pertinent to the secondary consequences of malnutrition and respiratory failure [11–13]. At present, there is not a definitive diagnostic test or biomarker available to identify ALS [14]. In this manner, neurologists rely solely on clinical criteria for diagnosis. Currently, some studies aim to find characteristics that address, in a more transparent and specific way, its etiology [15]. Thus, it is increasingly recognized the importance of preparing records that optimize the search for information for the clear and precise definition of aspects related to diseases considered rare and ALS.

For several years, researchers have seen the need and importance of implementing a clinical database that can provide support for the advancement of research in ALS [16–19]. Population studies have revealed ALS frequency in different continents and ethnicities. The significance of population registers is being increasingly observed as an essential complement to improve clinical assessment techniques [1, 20, 21]. The main constraint for the number of studies developed in the general ALS population and its subgroups is due to patients recruitment. Precisely, it means there are not enough accessible patients who meet the enrollment criteria, which requires collaboration amidst countries and continents to ensure a sufficient number of eligible individuals [22].

A registry is a system capable of collecting clinical and/or demographic data in an organized and safe way for a given purpose, using standardized observational study methods [23]. The main objective of the records is to capture all of the most significant numbers of cases of patients diagnosed with a specific pathology, regardless of age, health status, or socioeconomic status [24]. When analyzed, the captured data can provide information on the demographic characteristics of those diagnosed, effective monitoring of temporal and geographic trends in the distribution of the disease, and an investigation of environmental risk factors for diseases considered rare [25].

The results of these records should be utilized to promote health policies and allocate resources more efficiently [9]. Fundamentally, this is a public health issue that inevitably requires an organized and systematic response, which ought to include accurate data for surveillance and monitoring, as well as for individual care. To obtain more reliable statistics on the incidence and prevalence of rare diseases in each country and to facilitate applicable therapeutic translational research, the development of Rare Disease Registries (RDRs) is central to the solution of the problems presented [26–28]. RDRs include a diverse range of functionality, operate in different software environments, often incompatible, and serve a variety of purposes. In this way, Bellgard et al. [29] proposed a checklist for the development of RDRs involving the following aspects:

- Technological choices: definition of the type of platform, programming language, database, and configuration of the system deployment environment.
- System design: definition of the criteria for customization and modularization of the system.
- Software development: definition of the project manager and the development team, code versioning, deployment instructions, and system tests.
- Sustainability: data sharing must be simple, and it is essential to list all the effort demanded to maintain the registry.
- Interoperability: the register must have communication standards with other systems (through web services or ontologies).
- Security: two-step authentication process, users with variable access levels, data encryption and patient data anonymization process.
- Open source: enhanced levels of documentation, community feedback strategy, transparent and open installation process, and detailed deployment process.

This checklist can assist in defining the main criteria for a robust and sustainable implementation of specific RDR for a given disease.

### Objective

In view of the above scenario and considering that ALS is a rare and non reportable disease in much of the world, this article aims to identify the guidelines commonly applied on the structuring process of population records of rare diseases, focusing on Amyotrophic Lateral Sclerosis and its variations, to support the development of a national ALS registry in countries that do not yet have epidemiological data on the disease.

## Methods

This research was developed based on the systematic review guidelines proposed by Kitchenham [30] and following the preferred report items for systematic reviews and meta-analyses (PRISMA) [31], which consists of an evidence-based minimum set of artifacts for reporting in systematic reviews and meta-analyses. These resources were used in order to assist in the organization and understanding of the structure of population records related to Motor Neuron Diseases (MND) through the most recent publications in the literature that report the creation and design of rare disease registration models.

We analyzed the chief types of data comprised of those registers for its interoperability and data forwarding strategies. To this extent, major Research Questions (RQ) were listed to extract, by proper means, relevant information from the articles included in this systematic review. These RQ were essential for guiding the identification of the studies.

The RQ01 seeks to identify which patient data is collected in the national registry, focusing on the data used in each registry. It is necessary to be aware of what data is important to contribute to the purpose of the record. The RQ02 sought to identify the necessary data model for the development of the electronic registry. This RQ seeks to identify how records structure the collected data set. The RQ03 aimed to detect how such data is provided and made available for research. This way, we know how researchers can gain access to data after compilation. The purpose of the RQ04 is to identify the recruitment procedure, the data collection process, and the target audience for records. Based on these answers, we can identify whether the study was aimed at neurologists or at the patients themselves through self-registration. RQ05 aims to observe the level of interoperability of systems, detecting the existence of information exchange with records and other external systems. Finally, the last research question, the RQ06, sought to identify how the patient’s consent is made regarding the storage of their data in the national registry. It is essential to know the patient’s permission to use their data for research.

**Fig. 1:**
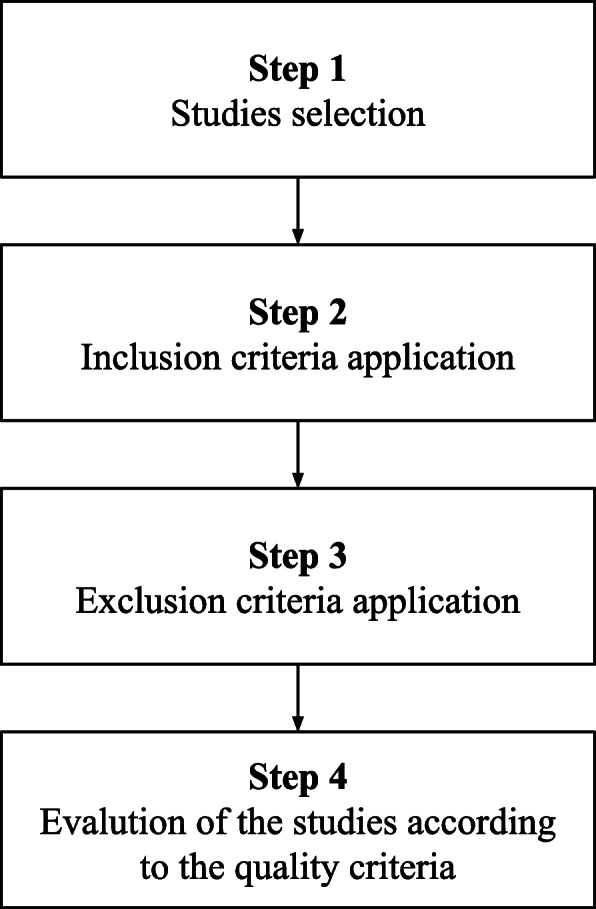
Protocol for execution of the systematic review

To develop and select studies, we designed a protocol to conduct this systematic review, as summarized in Fig. 1. Step 1 represents the selection of studies in the databases; step 2, the application of inclusion criteria; step 3, the application of exclusion criteria; and the step 4, the evaluation of articles according to quality criteria.

In the first step, we conducted bibliographic research in the following online databases: PubMed - U.S. National Institutes of Health’s National Library of Medicine, Scopus, ScienceDirect, SpringerLink. Moreover, the search query strings were: (“motor neuron disease” OR “ALS” OR “amyotrophic lateral sclerosis”) AND (“registry”). We only searched for studies in the English language.

Succeeding the search for the articles, it was necessary to filter those with a focus on the objective we proposed. Thus, defining criteria is fundamental for the carried out research, since the automated search engines might occasionally consider studies that are not related to the context of interest. Additionally, it possibly mitigates issues related to the selection of articles by keywords with semantics distinct from that established for investigation. Once selected in the previous step, the articles were analyzed based on the inclusion and exclusion criteria (Table 1).

**Table 1:** Inclusion and exclusion criteria applied to the searched studies

Inclusion criteria (IC)	Exclusion criteria (EC)
**IC01** - Articles published from January 2015 to September 2019;	**EC01** - Secondary studies or segments of the same research project;
**IC02** - Articles published in English;	**EC02** - Duplicate articles;
**IC03** - Articles that propose/report models, interoperability, guidelines, or transparency in the register and national monitoring of motor neuron diseases.	**EC03** - Book chapters or conference abstracts.

In the second step, year, language, and line of research were examined and included in this review in case such aspects met the inclusion criteria, presented in Table 1. These criteria contribute to a more comprehensive analysis of the set of articles returned from the listed databases, delimiting, with more specificity, the desired works. Subsequently, in the third step, the title, content of the abstract and keywords of articles were analyzed to exclude those that met the exclusion criteria highlighted in Table 1. With the smaller set of articles, these criteria contribute to a more detailed analysis, focusing on selecting articles that have relevant information for this systematic review.

After filtering the previous steps, the selected articles were analyzed in more depth. The fourth and ultimate step consisted of a Quality Assessment (QA). The QA was defined to analyze the relevance of the chosen research about the main objective of this systematic review. Other aspects analyzed were the methods and results achieved by these studies. The criteria selected to assess the quality of the studies took into account the points listed in Table 2. After the reading process, we assigned scores according to each criterion, based on the content of the articles, as follows:

1 $$ \begin{aligned} {\text{QA}} = \left\{ \begin{array}{ll} 1.0 & \textrm{, yes, the article completely approaches the subject},\\ 0.5 & \textrm{, the article partially approaches the subject},\\ 0 & \textrm{, the article does not approach the subject}.\\ \end{array} \right. \end{aligned}  $$

**Table 2:** Quality Assessment

QA	Description
01	Does the study precisely describe what data are available in the national registry?
02	Does the study describe how the data available in the national registry are accessed?
03	Does the study describe how data are forwarded to the national registry?
04	Does the study inform which technologies were utilized to develop the system?

From such an evaluation, it was generated the arithmetic mean of the score from the QA criteria for each article. Then, all articles that had a score greater than or equal to 0.5 (0.5≤*score*≤1) were selected for this research. Those also formed a set of pertinent studies that expose information regarding epidemiological records on neurological diseases. Consequently, they respond to the research questions previously prepared.

## Results

**Fig. 2:**
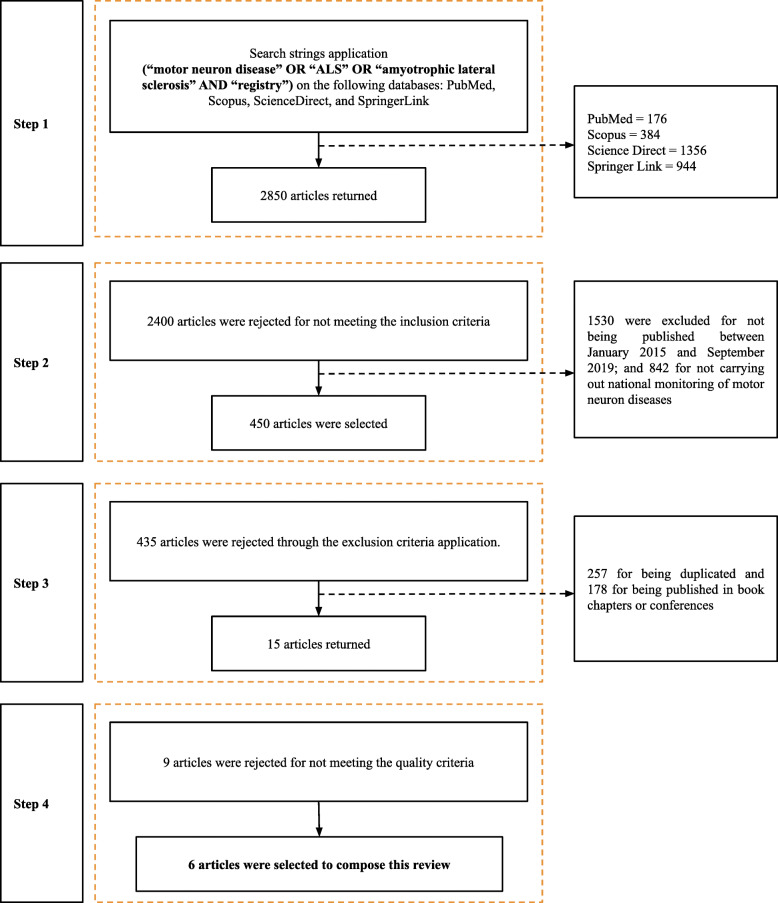
Execution of the study selection protocol in the systematic review

By following the previously detailed protocol, we initiated the phase of selecting the studies throughout September 2019. Thus, the phase consisted of applying the planned search strings on the databases. Figure 2 describes the execution of the study selection protocol.

The execution of the first step returned a total of 2850 studies. The Rayyan [32] tool was utilized to properly organize the extensive number of returned articles, assisting the development of posterior stages as well. After the execution of the second stage, 2400 papers were rejected since they did not meet the inclusion criteria. Of those, 1530 were excluded for their publication date discrepancy or for not reporting interoperability models, guidelines, or transparency in the registry. Still, 842 studies were also excluded for not performing national monitoring of motor neuron diseases.

By the conclusion of the second step, 450 articles were considered suitable for the subsequent one. In the third step, 435 articles were rejected according to the exclusion criteria: 257, for being duplicated, and 178, for being published in book chapters or conferences. After completion of such a stage, 15 articles were selected for quality assessment. With reference to the quality assessment step, nine studies were excluded for not providing fundamental information and, consequently, for not obtaining a suitable average score. To finalize the execution of the research protocol, six articles were included in the analysis and extraction of data according to the RQ.

### Analysis of selected studies

**Fig. 3:**
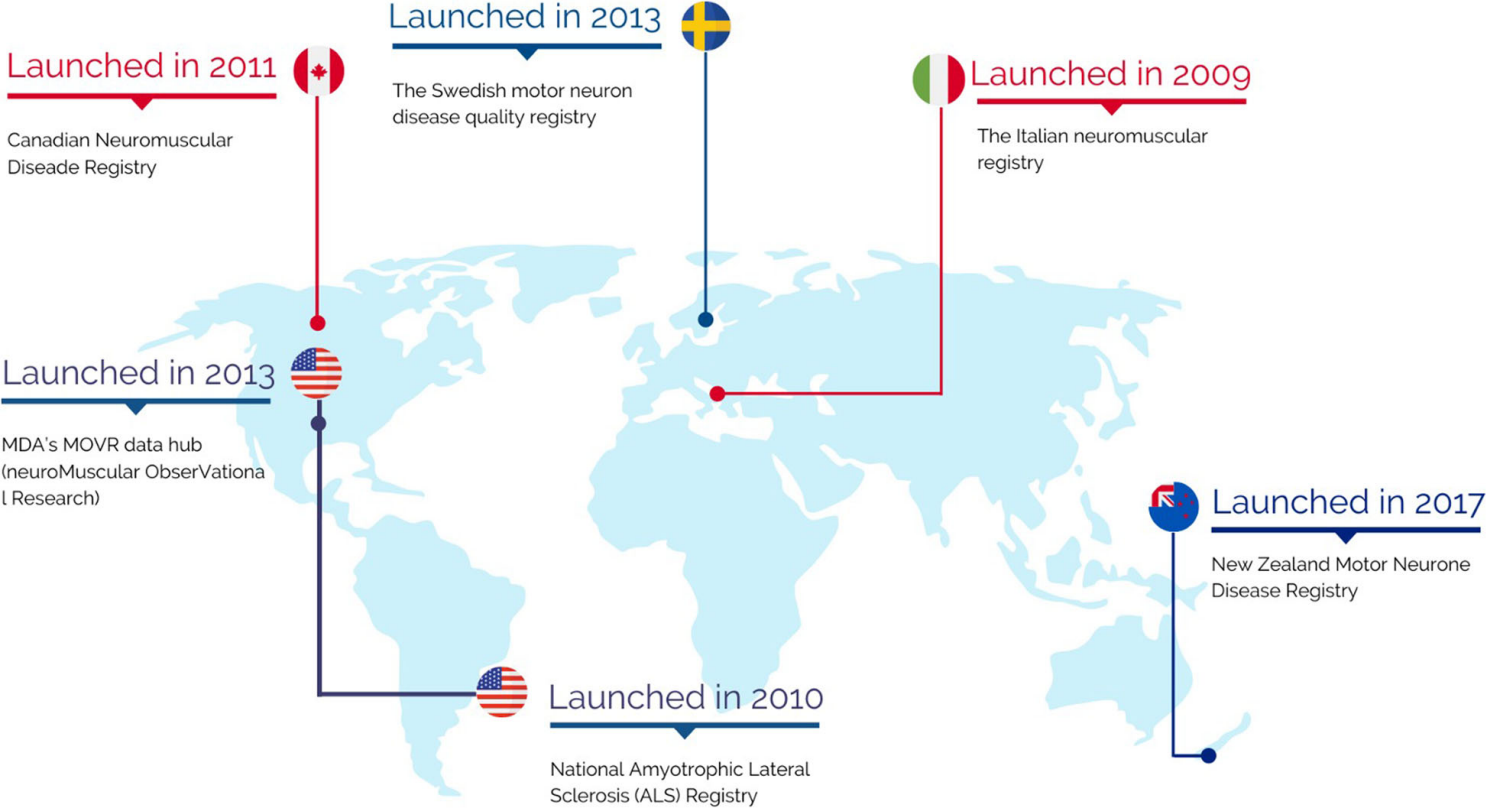
Map of the main registries developed between the years 2010 and 2017 by the literature. Figure 3 was produced by the authors using canvas (https://www.canva.com/). Images are available for free Canvas for Education

Overall, we selected five registers: two of which were located in Europe; three, in North America; and one, in Oceania (see map in Fig. 3). The registers presented were launched between the years 2010 to 2017.

Mehta [33] described the impacts, challenges, and subsequent directions of the National ALS Registry in the United States. In collaboration with the Centers for Disease Control and Prevention (CDC), this national registry was launched in October 2010. Its primary goals are presenting the incidence of the prevalence of the disease in the US, describing the demographics of people living with the condition, and examining risk factors associated with ALS development. Repository data access services are provided free of charge to researchers.

The authors present two strategies of patient recruitment: the search in an existing administrative database and the self-identification through the registry web portal. For ensuring a more precise determination of the collected data, the registry includes an algorithm that assists in the patients’ recruitment process, found in the analyzed databases. Those diagnosed with ALS are directly included in the registry, and patients who present characteristics for a possible diagnosis are not added. However, the data are retained in an administrative database so that the correct determination can be carried out in subsequent years. This registry collects demographic data, medication, risk factors, health care, and medical assistance. The patients without ALS characteristics or diagnosis are excluded from the registry.

The utilized algorithm was developed based on diagnostic code variables from International Classification of Diseases, Ninth Revision (ICD-9) related to ALS, neurologist visit frequency, and prescribed drugs. The other registration strategy is performed through online questionnaires, in which individuals inform their health status. These responses are analyzed to determine the cases considered ALS. In cases of use of both strategies by the same patient, the registry resolves the conflict by unifying the data and excluding existing duplicity. Repository data access services are provided free of charge to researchers. The register was developed with a view to future cooperation with the National ALS Biorepository, facilitating the exchange of data and information between the system. Besides, the registry utilizes anonymization techniques, anonymous data identification, and Global Unique Identifiers, allowing researchers to track patients’ progress in multiple studies in a secure and anonymized manner.

The Canadian Neuromuscular Disease Registry (CNDR), described by Wei et al. [34], was established in 2011 to improve the future of patients with neuromuscular diseases through training and support for research into possible treatments. The CNDR collects specific medical data on four rare pediatric neuromuscular diseases, collectively known as ’indexed’ diseases: Duchenne Muscular Dystrophy (DMD), Myotonic Dystrophy (DM), Spinal Muscular Atrophy (SMA), and Limb-Girdle Muscular Dystrophy (LGMD). In addition to these, the registry included other neuromuscular diseases. Patients are registered by their physicians and coordinating researchers in routine clinical visits. Data is updated at a minimum interval of once every 12 months. Patients who do not attend clinical sites unaffiliated with the CNDR, or who do not receive specialist care, can participate in the research by contacting its national office. Doctors can associate their patients with the registration by contacting the national office of the CNDR. In addition, data clinical and demographic data are collected retrospectively by trained researchers who access the clinic map, by the assistant physician, or by a trained research coordinator during a visit to the clinic. The identified and anonymous data are entered directly into a secure web portal.

The register’s proposal is in agreement with the European Alliance of Neuromuscular Disorders Associations (EAMDA). It is possible to ask for the register’s data through a formal application. There are two forms of solicitations: research proposal requests and statistical data requests. Proposals can be submitted by an investigator or a sponsor, being thus reviewed by the CNDR board committee. In summary, data is exported so as not to identify the patients. The CNDR provides a platform through which target populations can be rapidly and easily identified in a national complement of clinics, facilitating research activities. By the utilization of the register’s resources, it has been possible to connect Canadian patients with Neuromuscular Diseases (NMD) to national and international clinical trials. Hence, the CNDR acts as a valuable tool, allowing the capture of national data and facilitating a network of geographically diverse teams to help correct this gap, and serving as a basis for the development of registers of other NMD.

Concerned with essential governance resources to the proper functioning of the register, Ambrosini et al. [35] described the Italian neuromuscular disease Registry by analyzing its structure. Furthermore, the authors highlighted the scope of certain neurodegenerative disease registries in the international context. The registry is a platform that stores distinct databases for specific disease’s registration. Since this model can be employed to store data on several rare diseases, each register contains its own set of data to be collected. All clinical data collection protocols derive from international consensus or include standardized disease-specific data sets. Therefore, all records can share metadata and are potentially interoperable with their international efforts. Individuals who wish to participate in the research and live with a rare disease condition can register their personal data and complete dedicated surveys or Patient-Reported Results (PRR). This registry focuses on the collection of demographic data and general health history.

The completion of personal information generates a pseudo-minimization code (ID), which will later be attached to any data collected. The individuals select a preferred clinical center if required by the specific register so that doctors can collect and validate the medical data. The scientific coordinator/curator supervises the registration activity and accesses the data for ultimate validation and analysis. To ensure the security of the compiled material, the registry adopts some measures for data management, such as security and multi-level access for patients, specialists, and operators; separate compartments for storing personal patient’s information, with automatically generated pseudonymization codes; data encryption applied at varying levels; and an application and software database (data storage) hosted on dedicated virtual servers provided by a cloud service. Further, data can be utilized for publications, clinical trials, and data sharing with other entities.

The Italian NMD Registry may be considered as a flexible platform with an efficient structure to manage various specific databases developed according to the disease’s necessities. The implementation of this structure enables stakeholders to participate in its management with evident roles and responsibilities, contributing to the success of the research.

The NeuroMuscular ObserVational Research (MOVR), reported by Howell and Zuchner [36], consists of a register whose key objective is to gather evidence that can be used in current studies and experiments. The register includes information on demographic data, diagnostic test results, standardized measures of muscle function and health status, other clinical metrics, and records of medical interventions from more than 25 centers of care in the United States. MOVR data are collected by medical professionals at the service center site, ensuring the complete and accurate capture of highly detailed medical information. The focus of the record is to store data from patients with one of four neuromuscular diseases: ALS, DMD, SMA and Becker Muscular Dystrophy (BMD). It is significant to highlight that such a register combines individuals’ medical and genetic information, when available. The register is updated at each visit to the MDA Care Center, providing crucial continuity and allowing the longitudinal to record the symptoms. Researchers can employ the data to facilitate research by submitting an access request, which describes the proposed questions to be addressed, as well as their essentialism.

The study declared a commitment to work with other existing patient registries and share infrastructure insights and knowledge to improve the technology currently under development. The article suggests the classic approach to patients’ registers combined with internet technologies, data sharing platforms, electronic medical records, and, possibly, the most recent applications as artificial intelligence. Hence, such a tool will be ideal for associating research and clinical efforts, which usually are placed in distinct spaces.

The Swedish quality register for MND, described by Longinetti et al. [37], was launched in 2015. Its purpose is to ensure early diagnosis and high-quality medical care for all NMD patients (mainly from ALS) and create a research base for prospectively tracking the entire NMD population in Sweden. Clinical data are entered into the registry through follow-up visits every 12 weeks. Patients are informed of the inclusion of their data and may revoke it with a formal objection. Additionally, a committee of expert ALS doctors elected the registration data set. Separated by groups, the data set presents more than 100 variables, in which some are characterized as mandatory, forming the minimum register data set. It is also fed back by patients included in the register through a self-reported questionnaire. The information is evaluated by the health professional during follow-up visits. As a result, the completion of the questionnaire represents the attainment of most variables of the minimum set for entering patient data in the registry.

A managing committee decided on seven measures for patient self-reporting, including the Hospital Anxiety and Depression Scale (HADS) [38], self-reported use of other medications, Revised Amyotrophic Lateral Sclerosis Functional Rating Scale (ALSFRS-R) [39], pain classification on the Visual Analog Scale (VAS) [40], Life Satisfaction Questionnaire (LiSat-11) [41], Eating Assessment Tool (EAT-10) [42], and Body Mass Index (BMI) [43].

The authors also point out that the chief strengths of the registry consist of the wealth of data of its clinical, quantitative, qualitative, and prospective nature. Consequently, researchers are equipped with possible means of identifying appropriate candidates for clinical trials and other research projects, consistently providing the welfare and active participation of patients.

Given the scarcity of data on motor neuron diseases in New Zealand, Walker et al. [44] proposed the development of the New Zealand Motor Neurone Disease Registry (NZ MND), based on experiences and proposals already implemented in other reference registries already developed. The objective of the registry is to facilitate patient participation in research and clinical trials and assist researchers in planning and recruiting patients diagnosed with MND.

The NZ MND utilizes the same minimum data sets as the Australian Motor Neurone Disease Registry (AMNDR), which was also modeled for alignment with other international MND registries. Additional forms have been created or edited to comply with New Zealand prerequisites. The data reported in the system are related to demographics, medical data from the patient’s electronic record (through the National Health Index number), and genetic test data. A registry curator has been appointed to register participants, collect and enter data, ensure the achievement of regulatory requirements, and maintain data quality to meet daily registry execution. All identifiable data are maintained securely at the Auckland District Health Board in Auckland, New Zealand, where the registry is operated. Moreover, the anonymous clinical data are gathered in a cloud-based system hosted by Barwon Health in Geelong, Australia. It is possible to upload anonymized data to assist researchers’ study plans.

Participation in the NZ MND Registry is voluntary. Thus, patients’ recruitment occurs through a variety of methods, including a public webpage hosted by NZ MND, regular notices, messages sent to members, recruitment by the support staff, own references, and clinical references. The authors also address the importance of registration data being aligned with international standards, aiming at better data sharing and future cooperation. When implemented, the registry presented a data structure of considerable relevance. It may contribute to the advancement of research on neurodegenerative disease.

The researches found in the literature are composed of similar objectives: utilization of the most quality metrics and approaches for the development and management of a rare disease registry. Despite this similarity, all researches contain distinct strategies for achieving the proposed objective. Table 3 presents a brief comparison of the main pertinent aspects of each registry.

**Table 3:** International aspects of electronic registries for patients with motor neuron diseases

Register	Inte	Data capture	Self-registration	Data model	Patients’ recruitment	Disease
American register [33]	Yes	Web system	Yes	Not specified	Personal identification and database search	ALS
Canadian Neuromuscular Disease Registry (CNDR) [34]	No	Web system	No	TREAT-NMD	Patients from affiliated clinics	DMD, DM, SMA e LGMD
Italian neuromuscular registry [35]	Yes	Web system and clinical interview	Yes	TREAT-NMD	Platform’s disclosure and patients from clinics	DMD, BMD, SMA, CMT, MGSD, SBMA e TTR - FAP
NeuroMuscular ObserVational Research (MOVR) [36]	No	Visit to the MDA Care Center	No	Not specified	Medical indication	ALS, SMA, DMD e BMD
Swedish motor neuron disease quality registry [37]	No	Web system	Yes	Medical consensus	Analysis of hospital records	ALS
New Zealand Motor Neurone Disease Registry (NZ MND) [44]	No	Web system	No	AMNDR	Registry disclosure, advertisements, and medical indication	MND

**Abbreviations**: Inte = Interoperability, Cap. Dados = Captura dos dados, TREAT-NMD = Translational Research in Europe for the Assessment and Treatment of Neuromuscular Disease, AMNDR = Australian Motor Neurone Disease Registry, EUReMS = European Register for Multiple Sclerosis, ASL = Amyotrophic Lateral Sclerosis, DMD = Duchenne muscular dystrophy, SMA = Spinal Muscular Atrophy, CMT = Charcot-Marie-Tooth disease, BMD = Becker Muscular Dystrophy, MGSD = Muscle Glycogenoses, SBMA = Spinal-Bulbar Muscular Atrophy, TTR-FAP = Transthyretin-related familial amyloid polyneuropathy, DM = Myotonic Dystrophy, LGMD = Limb-Girdle Muscular Dystrophy, CNDR = Canadian Neuromuscular Disease Registry.

## Discussion

During protocol application, many studies were excluded for being out of the scope of this research and not describing models of registers. Predominantly, they solely mention their use for epidemiological studies, which does not represent the focus of this review. As few registers specify the contained data, there is an evident contrast between the oldest and newest concerning data quantity and quality. Despite the modest number of articles selected in this review, it is possible to observe, through the reported models, a panorama of the main necessary information to compose a national register for motor neuron disease.

The analyzed studies focused on the perspective of obtaining the data to characterize the researches of epidemiological studies. According to the analysis in Table 4, it is noticeable that the main data collected were demographic (ID01), present in all registers, and general health history (ID02), present in 50% of those analyzed.

**Table 4:** Main data categories identified in registers of motor neuron diseases

	American Registry [33]	CNDR [34]	Italian Registry[35]	MOVR [36]	Swedish Registry [37]	NZ MND [44]
ID01	∙	∙	∙	∙	∙	∙
ID02			∙		∙	∙
ID03					∙	
ID04					∙	
ID05					∙	
ID06				∙	∙	
ID07					∙	∙
ID08				∙	∙	
ID09				∙	∙	
ID010	∙				∙	
ID011					∙	
ID012					∙	
ID013					∙	
ID014	∙				∙	
ID015	∙			∙		

**Subtitle**: ID01 - Demographic data; ID02 - General health history; ID03 - Events related to diseases/injuries, Classification; ID04 - Pulmonary function test/respiratory status; ID05 - Vital signs and other bodily measures; ID06 - Physical and neurological exams; ID07 - Tests of laboratory and bio-specimens/biomarkers; ID08 - Imaging diagnostics; ID09 - Non-imaging diagnostics; ID10 - Medication; ID11 - Cognition; ID12 - Functional status; ID13 - Quality of life and mental health; ID14 - Risk factors; ID15 - Health Care and medical assistance.

Registries such as those from New Zealand [44] and Canada [34] focus on promoting clinical trials. As for the Swedish registry [37], it is more focused on building a register that presents detailed and quality data, not only generating data for research but also monitoring and improving the patients’ quality of life. These registers enable greater progress identification, follow-up, and aid in decision-making concerning health governance in line with trends that consider issues, as alignment with key international initiatives; ethics committee to evaluate research requests; register data curator; data confidentiality, ownership, and privacy; search for data in existing databases; development of a patient-oriented research-oriented register and algorithms for data validation [45]. Such questions indicate how important it is to align the development of registries with actions recommended by the World Health Organization (WHO).

The strategies of information capture are diverse. It is possible to perceive their functioning is immensely associated with the health system of each country. For this reason, distinct models are found, but that present similar approaches such as data capture in several layers, stages, and phases—being each related to particularities of the health system.The implementation of a web portal for patient self-reporting was informed in 50% of the cited records. We identified two ways of access, which can be: (1) open to anyone or (2) restricted to users who are previously registered by a health professional. In the American register, developed by Mehta [33], algorithms are used to verify data quality and veracity by comparing them with those from distinct sources. Such a factor points out the importance of identifying those that can feed register data. However, it has been assumed that utilizing web-based methods represents a barrier for the elderly, financially disadvantaged, technically inexperienced, or cognitively impaired.

In almost all registers, we verified the existence of access through the request for controlled access by an ethics committee associated with local entities or a professional trained and qualified exclusively for this activity. This process reinforces the importance of ethically evaluating the motivation of research and data use, an increasingly evident subject as one of the challenges of the 21st century. Nonetheless, we presume such a process tends to bureaucratize and hinder transparent and effective access.

International and national initiatives are always approached and debated for designing the registers. Such a fact demonstrates how the entities have been aware of the predominant models. Registers as the Italian [35] and Canadian [34] mention the Translational Research in Europe for the Assessment and Treatment of Neuromuscular Diseas (TREAT-NMD) network. Its main goals are developing a set of standardized principles and representing a consistent framework for recording patients with neuromuscular diseases.

Developed by Longinetti [37], the Swedish register introduces innovative practices and approaches, such as the use of a minimum data set for validation of patient information; a series of evaluation steps; data capture in stages; and the development of patient-centered records. Such practices aim at improving the quality of the register, ensuring its continuous success, and reinforcing closer interaction with the patient, which is not evident in other platforms. Additionally, the user-centered design strengthens the interaction between patients and ALS clinical team. Participants can also receive constant feedback about data for personal use and self-monitoring, contextualized across the population of the study. This feedback is used to request the indication of any DNM, acting as a channel for consulting the registry and actively seeking studies for patient participation.

The development of robust, interoperable, safe registers, that generate value for research and the patient, poses numerous challenges. In this research, even with the small number of studies selected and analyzed, it is possible to observe the trends and established strategies of registries worldwide. Despite international efforts and initiatives that have been mobilizing the creation of registries for motor neuron diseases, the alignment of the data has not yet identified a well-defined global set. Data standardization initiatives are already available and have been extensively discussed in the presented registries. Nonetheless, they are still unconsolidated. Furthermore, it is essential to intensify these standardization strategies when designing new registers.

Multiple actions are necessary for a population register of motor neuron diseases to achieve success. Hence, future resources should favor fundamental aspects such as establishing a coherent capture method with national health governance efforts; defining feasible national and international collaborations for data sharing; identifying the manager/curator or data controller based on precise definitions of governance structures; as well as acting in compliance with local data protection.

New patient-focused registry initiatives, which attempt to generate more value for patient care and treatment, trigger a motivation to implement other new national registries aimed at solutions beyond the most common primary causes, such as epidemiology, research development, and promotion of clinical trials. Fortunately, it is possible to design the creation of more robust and sustainable registers that will come to assist in capturing vital information as the understanding of disease processes improves immensely—through fruitful advances in data capture and science strategies. Captured data can not only direct research and development but also improvements in clinical care, policies, and results across the population for all people with motor neuron diseases.

## Conclusion

This systematic review aimed at identifying a population register panorama for motor neuron diseases, with a focus on aspects as data, technologies, access strategies, and data forwarding. Through this research, it was possible to detail the most effective practices, the protocols to be followed, the data models adopted, the security techniques, and the form of data interoperability.

The development of national registries for the monitoring and follow-up of MND depends on the contributions from diverse fields and engagement to promote partnerships between local entities. Moreover, their models considerably vary depending on the specificity of each location. Scientific societies and health institutions may fulfill a significant role in raising awareness and disseminating registries on motor neuron diseases through education and training. They must become, thus, more proactive in providing advice to policymakers.

The scarcity of studies on the incidence and prevalence of MND in the world is an indicator of the deficiency of consistent data on these diseases. Such a fact exerts decisive effects on the conduct of supplemental public policies, suggesting the need for research in this field. Further, the development of robust, interoperable, and secure electronic registries that generate value for research and patients presents itself as a solution and a challenge. This systematic review demonstrated the success of a population register requires actions with well-defined development methods, as well as the involvement of various actors of civil society.

## Data Availability

Not applicable.

## Abbreviations

ALS: Amyotrophic Lateral Sclerosis
RDRs: Rare Disease Registries
MND: Motor Neuron Diseases
RQ: Research Questions
QA: Quality Assessment
CDC: Centers for Disease Control and Prevention
ICD-9: International Classification of Diseases, Ninth Revision
DMD: Duchenne Muscular Dystrophy
DM: Myotonic Dystrophy
SMA: Spinal Muscular Atrophy
LGMD: Limb-Girdle Muscular Dystrophy
EAMDA: European Alliance of Neuro-muscular Disorders Associations
NMD: NeuroMuscular Diseases
PRR: Patient-Reported Results
MOVR: NeuroMuscular ObserVational Research
BMD: Becker Muscular Dystrophy
HADS: Hospital Anxiety and Depression Scale
ALSFRS-R: Revised Amyotrophic Lateral Sclerosis Functional Rating Scale
VAS: Visual Analog Scale
LiSat-11: Life Satis-faction Questionnaire
EAT-10: Eating Assessment Tool
BMI: Body Mass Index
NZ MND: New Zealand Motor Neurone Disease Reg-istry
AMNDR: Australian Motor Neurone Disease Registry
WHO: World Health Organization
PRISMA: Preferred Reporting Items for Systematic Reviews and Meta-Analyses
